# Analysis of Fatigue Crack Nucleation in Double-Network Hydrogels

**DOI:** 10.3390/polym16121700

**Published:** 2024-06-14

**Authors:** Shan Gao, Liying Jiang

**Affiliations:** Department of Mechanical and Materials Engineering, The University of Western Ontario, London, ON N6A 5B9, Canada; sgao283@uwo.ca

**Keywords:** double-network hydrogels, fatigue crack nucleation, configurational stress, microdamage mechanisms

## Abstract

Hydrogel-based devices commonly have a high demand for material durability when subjected to prolonged or cyclic loads. To extend their service life, it is crucial to have a deep understanding of the fatigue mechanisms of hydrogels. It is well-known that double-network (DN) hydrogels are characterized by high strength and toughness and are thus recognized as a promising candidate under load-bearing conditions. However, the existing studies in the literature mainly focus on their resistant capability to fatigue crack growth, while the underlying mechanisms of fatigue crack nucleation are still inconclusive. This work aims to bridge this knowledge gap by formulating a fatigue life predictor for DN hydrogels within the framework of configurational mechanics to elucidate the underlying mechanisms governing fatigue crack nucleation. The fatigue life predictor for DN hydrogels is derived from the configurational stress by incorporating the corresponding constitutive models and the thermodynamic evolution laws for microdamage mechanisms and material viscoelasticity. With the developed fatigue predictor, the effect of the microdamage mechanism on fatigue is elucidated, i.e., the internal damage of the sacrificial network can improve the fatigue life of DN hydrogels. The fatigue life predictor is also adopted to evaluate the effects of some other factors on the fatigue crack nucleation, such as the loading rate, pre-stretching treatment, and water diffusion, identifying feasible loading profiles that could improve material durability. Overall, the theoretical framework and the modeling results in this work are expected to shed light on unveiling the fatigue mechanisms of DN hydrogels and advance the design of hydrogel-based devices.

## 1. Introduction

Hydrogels, crosslinked polymer networks with swelling water, have attracted notable attention from the research community in recent years. The unique polymer–water structure endows hydrogels with high compliance and excellent biocompatibility, making them promising candidates in various fields such as soft robotics, wearable devices, tissue engineering, drug delivery [1,2,3,4], etc. With the proliferation of these real-world applications, one major concern has arisen regarding the durability of hydrogel-based devices, especially when subjected to prolonged loads and cyclic deformations.

Due to low strength, stiffness, and toughness [5,6], conventional single-network hydrogels are susceptible to fatigue and fracture, rendering them unsuitable for load-bearing applications. To improve the mechanical performance, various tough hydrogels have been developed and synthesized by introducing internal dissipation mechanisms, including double networks, hydrogen bonds, crystallinity, folded crosslinkers, fiber composites, and hierarchy structures, to name a few [7,8,9,10,11,12,13,14,15]. Notably, double-network (DN) hydrogels characterized by two interpenetrating polymer networks, i.e., the primary network is highly stretchable with loosely crosslinked long chains, while the sacrificial one is densely crosslinked with short chains, have sparked significant research interest. Such a unique structure equips DN hydrogels with enhanced resistance to fatigue and fracture [7,9,16]. When subjected to stretching, the sacrificial network damages and dissipates significant mechanical energy, while the primary network retains its structure, ensuring the integrity and elasticity of the hydrogel. In addition to the anti-fatigue and fracture properties, the microdamage of the sacrificial network also gives rise to the macroscopic Mullins-type response of DN hydrogels [5,17,18]. Furthermore, by introducing various types of crosslinks and network topologies, DN hydrogels are also endowed with diverse mechanical behaviors such as rate-dependent viscosity [17] and self-recovery capability [5,8]. Furthermore, the microdamage of the sacrificial network also affects the swollen state of DN hydrogels. Nakajima et al. [19] and Mai et al. [20] observed an anisotropic reswelling phenomenon on pre-stretched DN hydrogels, which is attributed to the anisotropic microdamage of the sacrificial network. Most recently, Imaoka et al. [21] first experimentally captured an extension-induced deswelling behavior of a highly deformed PNaMPS-PAAm hydrogel. 

Over the past decade, the research community has also devoted much effort to the theoretical modeling of the microdamage-induced behaviors of DN hydrogels. To establish the bridge between the macroscopic behaviors and the microscopic damage, a typical method is to incorporate damage variables into the free energy density functions and formulate the corresponding damage evolution laws. Wang and Hong [22] modified the Gent model [23] by introducing two damage variables, i.e., the damage degree and the stretching limit of the sacrificial network, associating the macroscopic Mullins effect and the neck instability of DN hydrogels with the microdamage of the sacrificial network in a phenomenological manner. Zhao [24] incorporated the network alteration theory [25,26] with the eight-chain statistical model [27] to reveal the effects of the microdamage mechanisms, i.e., the evolution of chain density and chain stretchability limit, on the Mullins-type behavior as well as the neck instability. Inspired by the work of Zhao [24], Mao et al. [17] adopted a Maxwell model with damageable springs and combined the Gent model and the network alteration theory to characterize the coupled Mullins effect and the ionic bonds-breaking-induced viscoelastic response. Meanwhile, Lu et al. [18] employed the Kelvin–Voigt model in tandem with the damage evolution law proposed by Ogden and Roxburgh [28] to model the viscoelastic and Mullins effects. Moreover, some other mechanisms are also constitutively described, like the concurrent viscoelastic, self-healing, and failure of a tough physical hydrogel [29] and the fatigue-induced stress-softening behaviors in multi-network elastomers and hydrogels [30,31]. These constitutive models were established on the basis of isotropic microdamage assumption and showed satisfactory agreement on the mechanical response of DN hydrogels. It should be noted that those constitutive models neglect solvent diffusion during the deformation process and take DN hydrogels as incompressible materials. The justification for such an assumption lies in the fact that the long-range transportation of solvent molecules takes a much longer time than the internal damage process. However, the solvent diffusion effect should be considered when the DN hydrogel has experienced a large pre-stretching and will undergo subsequent deformation in a solvent for a relatively long time. The pre-stretching treatment is expected to induce microdamage of the sacrificial network, allowing DN hydrogels to gain extra swelling in the solvent [19]. Liu et al. [32] provided a prediction of the anisotropic reswelling of a pre-stretched PAMPS-PAAm hydrogel [19] by using a micro-sphere network model where the anisotropy in the microdamage is naturally introduced. Whereas the pre-stretched PNaAMPS-PAAm hydrogel is slightly anisotropic, a recent work by Imaoka et al. [21] showed excellent agreement on the reswelling and the equilibrium swelling behaviors by assuming the isotropy of the polymer networks for simplification in modeling. Considering the good predictions on the mechanical response [17,18,22,24,29] and the subsequent swelling phenomenon [21], the isotropic microdamage assumption will be adopted in the current work.

Like other soft materials, hydrogels are often subjected to prolonged or cyclic deformation. Durability and fatigue analysis is a critical issue that needs to be addressed in order to fulfill their potential applications. However, there are few models in the literature for predicting the fatigue damage of hydrogels. In the literature, the fatigue life of materials is usually assessed through two approaches: crack nucleation and crack growth [33,34]. Stemming from fracture mechanics, the crack growth method has been widely used to study the fracture and fatigue failure of pre-cut DN hydrogels both experimentally [5,9,35,36,37,38] and numerically [39,40,41]. However, the application of the crack growth approach is contingent on knowing the up-front information of existing cracks, aiming to connect the fatigue to the crack’s initial geometry and energy release rate. To address this limitation, the crack-nucleation approach emerges as a viable alternative. The essence of the crack-nucleation approach lies in proposing a continuum-based quantity as the fatigue life predictor, which is capable of characterizing the fatigue damage contributing to crack nucleation. As commented upon in [33,34,42,43], the configurational stress tensor is able to quantify the energy that drives the crack nucleation, showing superiority in fatigue life prediction. It surpasses the conventional predictors, namely, the maximum strain, the maximum principal Cauchy stress, and the maximum strain energy density, especially in the context of unifying the multiaxial fatigue data and capturing the crack closure. In addition, various mechanisms that may dominate fatigue damage can be incorporated into the configurational mechanics framework—for example, electromagnetism [44], thermoelasticity [45], elastoplasticity [46], piezoelectricity [47], viscoelasticity [42], and coupled water diffusion–large deformation behavior of hydrogels [43].

Limited to the realm of hydrogels, our preliminary work [43] has derived the configurational stress tensor specific to neutral single-network hydrogels and discerned two origins of the fatigue damage, namely, the stretching of the polymer network and the mixing of a polymer network with water molecules. When it comes to DN hydrogels, few studies focus on the fatigue crack-nucleation process, and the impact of the microdamage mechanisms pertaining to the sacrificial network upon the fatigue crack nucleation of the DN hydrogels still remains unclear. In addition, the combined effects of microdamage, material viscosity, and swelling on the fatigue crack nucleation need further investigation. To bridge these knowledge gaps, we extend the configurational stress tensor to DN hydrogels and attempt to unveil the potential factors governing the fatigue crack nucleation of DN hydrogels. This work is expected to shed light on understanding the relation between the fatigue damage of DN hydrogels and the microstructural change in polymer networks.

The remainder of the paper is organized as follows. Section 2 outlines the constitutive theory of DN hydrogels and the formulation of the configurational stress tensor. In Section 3, two representative case studies are presented with the selected material models. Section 4 summarizes and concludes this research.

## 2. Formulation of the Configurational Stress Tensor

In this section, we will briefly review the constitutive theory of DN hydrogels with coupled nonlinear deformation and solvent diffusion behavior. Subsequently, we will present the formulation of the configurational stress tensor as the fatigue life predictor for DN hydrogels within the configurational mechanics framework.

### 2.1. Constitutive Theory for DN Hydrogels

For a body of stress-free and solvent-free (dry) DN hydrogel identified with an occupied region Ω of space in a fixed reference configuration, the position of an arbitrary material point in Ω is denoted as **X**. The material point motion from the reference state Ω to the current state $\Omega_{t}$ undergoing deformation and swelling is a smooth one-to-one mapping $x=\chi \left(X,t\right)$ with deformation gradient **F** defined as

(1) $$F=\nabla \chi =\frac{\partial x}{\partial X}$$

To account for the concurrent change of shape and volume of Ω, we adopt a multiplicative decomposition of the deformation gradient **F** into a mechanical part **F***_m_* and a swelling part **F***_s_* [48,49], i.e.,

(2) $$F=F_{m}F_{s}$$

Following the treatment of Duda et al. [48], the mechanical deformation **F***_m_* is considered as isochoric, so that the volume change of Ω is solely induced by the swelling process, leading to

(3) $$\det\left(F_{m}\right)=1,\det\left(F\right)=\det\left(F_{s}\right)$$

Owing to the presence of the sacrificial network, DN hydrogels exhibit a viscoelastic response. To characterize the viscous response, the mechanical deformation gradient **F***_m_* admits a further multiplicative decomposition into an elastic part **F***_e_* and a viscous part **F***_v_* [17,49], i.e.,

(4) $$F_{m}=F_{e}F_{v}$$

With the adoption of isotropic swelling assumption, the swelling component **F***_s_* can be expressed as

(5) $$F_{s}={\left(\det\left(F\right)\right)}^{\frac{1}{3}}I$$

**Figure 1:**
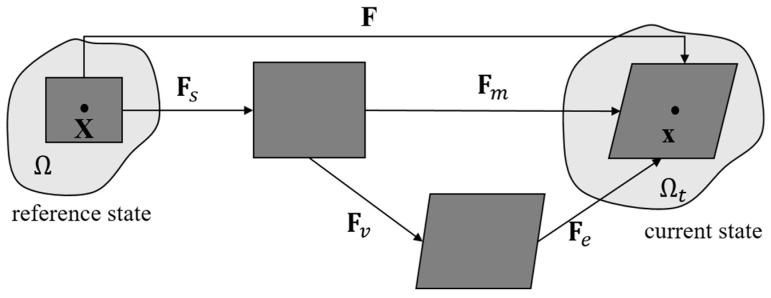
Multiplicative decomposition of the deformation gradient **F** into a swelling part **F***_s_* and a mechanical part **F***_m_* [48]. The mechanical deformation is further decomposed into an elastic part **F***_e_* and a viscous part **F***_v_* [17,49].

The entire multiplicative decomposition of **F** is illustrated in Figure 1.

For the hydrogel system, the force banlance in continuum mechanics dictates that the nominal stress **S** satisfies

(6) DivS+B=0 in Ω

(7) S⋅n=T on ∂Ω

where **B** is the body force per unit reference volume, and **T** is the nominal traction on the unit area of the boundary ∂Ω, with **n** being the unit vector normal to the boundary ∂Ω. Meanwhile, given that no chemical reactions occur within the hydrogel body, the mass conservation of the solvent molecules requires

(8) $$\dot{C}+\mathrm{Div}J=r\;\mathrm{in}\;\Omega$$

(9) J⋅n=−i on ∂Ω

Here, *C* is the nominal concentration of solvent molecules measured per unit reference volume, and **J** is the fluid flux measured per unit reference area, per unit time. *r* and *i* denote the number of solvent molecules per unit time injected into the hydrogel per unit reference volume and per unit reference area, respectively.

Thermodynamics dictates that the rate of the change of the total free energy δG/δt should never increase, i.e.,

(10) $$\frac{\delta G}{\delta t}=\int_{\Omega }\frac{\delta W}{\delta t}dV-\int_{\Omega }B\cdot \frac{\delta x}{\delta t}dV-\int_{\partial \Omega }T\cdot \frac{\delta x}{\delta t}dA-\int_{\Omega }\mu rdV-\int_{\partial \Omega }\mu idA\leq 0$$

where μ is the chemical potential of the solvent, and *W* is the Helmholtz free energy density of the hydrogel [50,51,52,53,54] described by

(11) $$W=\mu_{0}^{L}C+W_{\mathrm{stretch}}+W_{\mathrm{mix}}$$

$\mu_{0}^{L}C$ is the energy of solvent molecules not interacting with the polymer networks, and $W_{\mathrm{stretch}}$ and $W_{\mathrm{mix}}$ represent the stretching energy of the polymer networks and the mixing energy of the polymer networks and the interstitial solvent, respectively. The mixing energy $W_{\mathrm{mix}}$ is a function of the nominal concentration *C*. For DN hydrogels containing two polymer networks, the stretching energy $W_{\mathrm{stretch}}$ is defined as [41],

(12) $$W_{\mathrm{stretch}}=\sum_{i=1}^{2}W_{\mathrm{stretch}}^{\left(i\right)}\left(F,F_{e}^{\left(i\right)},\xi^{\left(i\right)}\right)=\sum_{i=1}^{2}\left[W_{\mathrm{stretch}}^{\mathrm{EQ}\left(i\right)}\left(F,\xi^{\mathrm{EQ}\left(i\right)}\right)+W_{\mathrm{stretch}}^{\mathrm{NEQ}\left(i\right)}\left(F_{e}^{\left(i\right)},\xi^{\mathrm{NEQ}\left(i\right)}\right)\right]$$

where the superscripts “EQ(*i*)” and “NEQ(*i*)” denote the equilibrium and the non-equilibrium energy of the *i*th polymer network, respectively. The internal variable $\xi^{\left(i\right)}$ characterizes the microstructural change in the *i*th polymer network during the deformation. The non-equilibrium part $W_{\mathrm{stretch}}^{\mathrm{NEQ}\left(i\right)}$ is associated with the viscoelastic response of the *i*th polymer network.

Combining Equations (6)–(9), (11), and (12), the inequality (10) becomes

(13) $$\begin{array}{l} \int_{\Omega }\left(\frac{\partial W}{\partial F}-S\right):\dot{F}dV+\int_{\Omega }\left(\frac{\partial W}{\partial C}-\mu \right)rdV+\int_{\partial \Omega }\left(\frac{\partial W}{\partial C}-\mu \right)idA\\ +\sum_{i=1}^{2}\int_{\Omega }\frac{\partial W_{\mathrm{stretch}}^{\left(i\right)}}{\partial F_{e}^{\left(i\right)}}\frac{\partial F_{e}^{\left(i\right)}}{\partial F_{v}^{\left(i\right)}}:{\dot{F}}_{v}^{\left(i\right)}dV+\sum_{i=1}^{2}\int_{\Omega }\frac{\partial W_{\mathrm{stretch}}^{\left(i\right)}}{\partial \xi^{\left(i\right)}}\frac{\delta \xi^{\left(i\right)}}{\delta t}dV+\int_{\Omega }J\cdot \mathrm{Grad}\left(\frac{\partial W}{\partial C}\right)dV\leq 0 \end{array}$$

According to Coleman and Noll [55], the constitutive relations are given as

(14) $$S=\frac{\partial W}{\partial F}$$

(15) $$\mu =\frac{\partial W}{\partial C}$$

Therefore, the inequality (13) is reduced to and rewritten as

(16) $$-\sum_{i=1}^{2}\frac{\partial W_{\mathrm{stretch}}^{\left(i\right)}}{\partial \xi^{\left(i\right)}}{\dot{\xi }}^{\left(i\right)}+\sum_{i=1}^{2}M_{e}^{\left(i\right)}:L_{v}^{\left(i\right)}-J\cdot \mathrm{Grad}\mu \geq 0$$

where $M_{e}^{\left(i\right)}=2C_{e}^{\left(i\right)}\frac{\partial W_{\mathrm{stretch}}^{\left(i\right)}}{\partial C_{e}^{\left(i\right)}}$ denotes the elastic part of the Mandel stress, and $L_{v}^{\left(i\right)}={\dot{F}}_{v}^{\left(i\right)}{\left(F_{v}^{\left(i\right)}\right)}^{-1}$ is the viscous velocity gradient for the *i*th network. To ensure the inequality of (16), the following conditions must hold true for arbitrary admissible process [17,56], i.e.,

(17a) $$-\frac{\partial W_{\mathrm{stretch}}^{\left(i\right)}}{\partial \xi^{\left(i\right)}}{\dot{\xi }}^{\left(i\right)}\geq 0\;\mathrm{for}\;\mathrm{each}\;i$$

(17b) $$M_{e}^{\left(i\right)}:L_{v}^{\left(i\right)}\geq 0\;\mathrm{for}\;\mathrm{each}\;i$$

(17c) −J⋅Gradμ≥0

The inequality (17a) represents the energy dissipation stemming from the internal damage of the sacrificial network. The specification of the damage variable $\xi^{\left(i\right)}$ and the corresponding evolution law will be discussed later in Section 3.1. The inequality (17b) represents the energy dissipation due to the viscous flow, which is commonly assumed as irrotational and incompressible [17,56,57], i.e.,

(18) $$W_{v}^{\left(i\right)}=0\;\mathrm{and}\;\det\left(F_{v}^{\left(i\right)}\right)=1$$

such that the evolution of $F_{v}^{\left(i\right)}$ follows

(19) $${\dot{F}}_{v}^{\left(i\right)}=D_{v}^{\left(i\right)}{\left(F_{v}^{\left(i\right)}\right)}^{-1}\;\mathrm{and}\;\mathrm{tr}\left(D_{v}^{\left(i\right)}\right)=0$$

with $W_{v}^{\left(i\right)}=\frac{1}{2}\left(L_{v}^{\left(i\right)}-L_{v}^{\left(i\right)T}\right)$ being the viscous spin tensor and $D_{v}^{\left(i\right)}=\frac{1}{2}\left(L_{v}^{\left(i\right)}+L_{v}^{\left(i\right)T}\right)$ being the viscous stretching tensor. According to the rate-dependent flow rule [17,57], the viscous stretching tensor $D_{v}^{\left(i\right)}$ is expressed as

(20) $$D_{v}^{\left(i\right)}={\dot{\gamma }}_{v}^{\left(i\right)}\left(\frac{M_{e0}^{\left(i\right)}}{2{\bar{\tau }}^{\left(i\right)}}\right)$$

Here, $M_{e0}^{\left(i\right)}$ is the deviatoric part of the elastic Mandel stress $M_{e}^{\left(i\right)}$ of the *i*th network, and ${\bar{\tau }}^{\left(i\right)}$ is the equivalent shear stress for each network with the definition of

(21) $${\bar{\tau }}^{\left(i\right)}=\frac{1}{\sqrt{2}}\sqrt{M_{e0}^{\left(i\right)}:M_{e0}^{\left(i\right)}}$$

and ${\dot{\gamma }}_{v}^{\left(i\right)}$ is the equivalent viscous shear strain rate described in terms of a power-law function, i.e.,

(22) $${\dot{\gamma }}_{v}^{\left(i\right)}={\dot{\gamma }}_{0}^{\left(i\right)}{\left(\frac{{\bar{\tau }}^{\left(i\right)}}{s^{\left(i\right)}}\right)}^{\frac{1}{m^{\left(i\right)}}}$$

where ${\dot{\gamma }}_{0}^{\left(i\right)}$ is the reference equivalent strain rate for the *i*th network, $m^{\left(i\right)}$ is a strain-rate sensitivity parameter, and $s^{\left(i\right)}$ is an internal variable accounting for the change in the resistance to the viscous flow. The inequality (17c) represents the energy dissipation due to the solvent diffusion. A common practice to satisfy the inequality of (17c) is to define the fluid flux **J** following a kinetic law [43,53], i.e.,

(23) $$J=-\frac{D}{vkT}F^{-1}F^{-T}\left(\det\left(F\right)-1\right)\cdot \mathrm{Grad}\mu$$

where *D* is the diffusion coefficient, *v* is the volume per solvent molecule, *k* is the Boltzmann constant, and *T* is the temperature.

For hydrogels, the coupled large deformation and solvent diffusion behavior is commonly restricted by the molecular incompressibility condition for both the polymer networks and the solvent molecules [43,53], leading to

(24) $$\det\left(F\right)=1+vC$$

Such a condition is enforced by adding an extra term of $\varpi \left(1+vC-\det\left(F\right)\right)$ to the free energy density Equation (11), with ϖ being the Lagrange multiplier. Correspondingly, the constitutive relations in Equations (14) and (15) are modified as

(25) $$S=\sum_{i=1}^{2}\left(\frac{\partial W_{\mathrm{stretch}}^{\mathrm{EQ}\left(i\right)}}{\partial F}+\frac{\partial W_{\mathrm{stretch}}^{\mathrm{NEQ}\left(i\right)}}{\partial F}\right)-\varpi \det\left(F\right)F^{-T}\;$$

(26) $$\mu -\mu_{0}=\frac{\partial W_{\mathrm{mix}}\left(C\right)}{\partial C}+v\varpi$$

Recall the definition of the liquid pressure *p* with $p=\frac{\mu -\mu_{0}^{L}}{v}$ and the osmotic pressure π with $\pi =-\frac{1}{v}\frac{\partial W_{\mathrm{mix}}\left(C\right)}{\partial C}$ [43,48,54], and the Lagrangian multiplier ϖ can be expressed as ϖ=p+π. Therefore, the nominal stress **S** as defined in Equation (25) can be rewritten as [54]

(27) $$S=\sum_{i=1}^{2}\left(\frac{\partial W_{\mathrm{stretch}}^{\mathrm{EQ}\left(i\right)}}{\partial F}+\frac{\partial W_{\mathrm{stretch}}^{\mathrm{NEQ}\left(i\right)}}{\partial F}\right)-\pi \det\left(F\right)F^{-T}-p\det\left(F\right)F^{-T}$$

As discussed in our previous work [43], the nominal stress in Equation (27) can be decomposed into a fluid stress ($-p\det\left(F\right)F^{-T}$) due to the pressurization of the interstitial fluid and a network stress **S***_n_* due to the distortion of the polymer networks, i.e.,

(28) $$S_{n}=\sum_{i=1}^{2}\left(\frac{\partial W_{\mathrm{stretch}}^{\mathrm{EQ}\left(i\right)}}{\partial F}+\frac{\partial W_{\mathrm{stretch}}^{\mathrm{NEQ}\left(i\right)}}{\partial F}\right)-\pi \det\left(F\right)F^{-T}$$

Such a network stress can be further decomposed into a stretching-induced stress $S_{n}^{\mathrm{str}}$ and a mixing-induced stress $S_{n}^{\mathrm{mix}}$, i.e.,

(29a) $$S_{n}^{\mathrm{str}}=\sum_{i=1}^{2}S_{n}^{\mathrm{str}\left(i\right)}=\sum_{i=1}^{2}\left(\frac{\partial W_{\mathrm{stretch}}^{\mathrm{EQ}\left(i\right)}}{\partial F}+\frac{\partial W_{\mathrm{stretch}}^{\mathrm{NEQ}\left(i\right)}}{\partial F}\right)$$

(29b) $$S_{n}^{\mathrm{mix}}=-\pi \det\left(F\right)F^{-T}$$

### 2.2. Fatigue Life Predictor for Crack Nucleation

**Figure 2:**
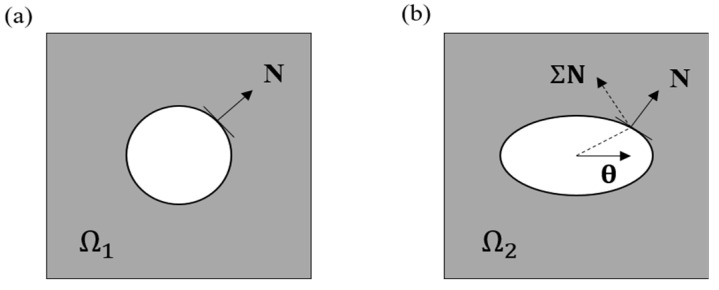
Evolution of a defect in a RVE from the reference state $\Omega_{1}$ (**a**) to the deformed state $\Omega_{2}$ (**b**). White area denotes the microscopic defect, and the gray area represents the bulk material.

From the perspective of crack nucleation, it has been widely believed that the nucleation of the macroscopic crack arises from the accumulation of the microscopic defects that naturally exist in the material [34,58]. The evolution of a defect is illustrated through a representative volume element (RVE), as demonstrated in Figure 2. When the material is subjected to any external load, the defect evolves (may grow or shrink) from its reference configuration $\Omega_{1}$ (Figure 2a) to a configuration $\Omega_{2}$ (Figure 2b). During this process, the irreversible evolution of the defects accumulates and eventually leads to crack nucleation. Such a crack-nucleation process is concomitant with energy release [43]. The configurational stress Σ [59,60] is thus considered as the driving force of the defect evolution due to its capability of characterizing such an energy change [43,61,62]. In Figure 2, the scalar θ⋅ΣN quantifies the change of the total energy, with θ and **N** being the material translation and the unit normal vector of the material surface, respectively. Considering that the hydrogel body tends to reduce as much energy as possible during the defect-evolution process, the direction of θ and **N** should be coincident with the principal directions of **−Σ** to maximize the released energy, i.e., $\theta \cdot \left(-\Sigma \right)N$. On this basis, the configurational stress is further extended as a predictor $\Sigma^{*}$ for the fatigue damage of hydrogels under cyclic load, which is defined as

(30) $$\Sigma^{*}=\left|\min\int_{\mathrm{cycle}}{\left(d\Sigma_{i}^{d}\right)}_{i=1,2,3}\right|$$

Here, we define ${\left(d\Sigma_{i}\right)}_{i=1,2,3}$ as the principal values of the increment in the configurational stress tensor dΣ i.e., $d\Sigma =\sum_{i=1}^{3}d\Sigma_{i}V_{i}⊗V_{i}$, with $V_{i}$ being the corresponding principal directions. According to Verron and Andriyana [34] and Gao et al. [43], the damage contributor $d\Sigma_{i}^{d}$ is determined when the increment in the principal configurational stress $d\Sigma_{i}$ satisfies the following conditions:

(31) $$d\Sigma_{i}^{d}=\left\{\begin{array}{ll} {d\Sigma }_{i} & \;\mathrm{if}\;d\Sigma_{i}<0\;\mathrm{and}\;V_{i}\cdot \Sigma_{i}V_{i}<0\\ 0 & \;\mathrm{otherwise} \end{array}\right.$$

Equation (31) indicates that the crack tends to nucleate only when the increment $d\Sigma_{i}$ opens a defect ($d\Sigma_{i}<0$) and the defect is in a materially stretched state in the direction of $V_{i}$, i.e., $V_{i}\cdot \Sigma_{i}V_{i}<0$.

According to our early work [43], the general expression of the configurational stress tensor for hydrogels was derived as

(32) $$\Sigma =\left(W-\mu C\right)I-F^{T}S$$

Substituting Equations (11) and (27) into Equation (32) and adopting the incompressibility condition of Equation (24), the configurational stress of DN hydrogels can also be decomposed into two parts:

(33) $$\Sigma =\Sigma_{n}+pI$$

where *p***I** is the configurational stress due to the fluid pressure, while $\Sigma_{n}$ is the configurational stress associated with the distortion of the polymer networks [43,54]. The network configurational stress is further decomposed to account for the contributions from stretching and mixing, i.e.,

(34a) $$\Sigma_{n}=\Sigma_{n}^{\mathrm{str}}+\Sigma_{n}^{\mathrm{mix}}$$

(34b) $$\Sigma_{n}^{\mathrm{str}}=\left(\sum_{i=1}^{2}W_{\mathrm{stretch}}^{\left(i\right)}\left(F,F_{e}^{\left(i\right)},\xi^{\left(i\right)}\right)\right)I-F^{T}\left(\sum_{i=1}^{2}S_{n}^{\mathrm{str}\left(i\right)}\right)$$

(34c) $$\Sigma_{n}^{\mathrm{mix}}=W_{\mathrm{mix}}I-F^{T}S_{n}^{\mathrm{mix}}$$

where $\Sigma_{n}^{\mathrm{str}}$ is the stretching-induced contribution, while $\Sigma_{n}^{\mathrm{mix}}$ is induced by mixing. According to our previous study [43], only the network configurational stress $\Sigma_{n}$ contributes to the fatigue damage of the hydrogels. Therefore, we will only keep the component of the network configurational stress in the fatigue predictor.

It is well adopted in the literature that for poro-visco-elasticity of hydrogels, viscosity is characterized with a short time scale, while water diffusion is a relative long-time-scale process [63]. In other words, when hydrogels exhibit viscosity, water diffusion can be ignored within a short-time period. While the polymer networks relax thoroughly and manifest insignificant viscosity when water diffusion in hydrogels becomes evident. Therefore, we treat viscosity and water diffusion as distinct processes when evaluating the corresponding fatigue damage. It was argued in the literature that for viscoelastic materials [34,42], only non-dissipative configurational stress contributes to crack opening or shrinking, which is derived as

(35) $${\left(\Sigma_{n}^{\mathrm{str}}\right)}^{ND}=\left(\sum_{i=1}^{2}W_{\mathrm{stretch}}^{\left(i\right)}\left(F,F_{e}^{\left(i\right)},\xi^{\left(i\right)}\right)\right)I-F_{s}^{T}\left(\sum_{i=1}^{2}F_{e}^{\left(i\right)T}S_{n}^{\mathrm{str}\left(i\right)}F_{v}^{\left(i\right)T}\right)$$

Correspondingly, with the consideration of the contribution to fatigue, the network configurational stress $\Sigma_{n}$ for DN hydrogels is modified as

(36) $$\Sigma_{n}={\left(\Sigma_{n}^{\mathrm{str}}\right)}^{ND}+\Sigma_{n}^{\mathrm{mix}}$$

Therefore, the crack nucleation criterion for DN hydrogels as defined in Equation (31) is further modified by replacing Σ with $\Sigma_{n}$, leading to

(37) $$d\Sigma_{i}^{d}=\left\{\begin{array}{ll} d{\left(\Sigma_{n}\right)}_{i} & \;\mathrm{if}\;d{\left(\Sigma_{n}\right)}_{i}<0\;\mathrm{and}\;V_{i}\cdot \left[{\left(\Sigma_{n}\right)}_{i}-{\left(\Sigma_{n}\right)}_{i0}\right]V_{i}<0\\ 0 & \;\mathrm{otherwise} \end{array}\right.$$

where ${\left(\Sigma_{n}\right)}_{i0}$ represents the initial value of network configurational stress ${\left(\Sigma_{n}\right)}_{i}$ at the starting point of the steady loading cycle, and $d{\left(\Sigma_{n}\right)}_{i=1,2,3}$ are the principal values of the increment of the network configurational stress tensor $d\Sigma_{n}$, i.e.,

(38) $$d\Sigma_{n}=d{\left(\Sigma_{n}^{\mathrm{str}}\right)}^{ND}+d\Sigma_{n}^{\mathrm{mix}}=\sum_{i=1}^{3}d{\left(\Sigma_{n}^{\mathrm{str}}\right)}_{i}^{ND}V_{i}⊗V_{i}+d\left(\Sigma_{n}^{\mathrm{mix}}\right)I$$

## 3. Representative Case Study

**Figure 3:**
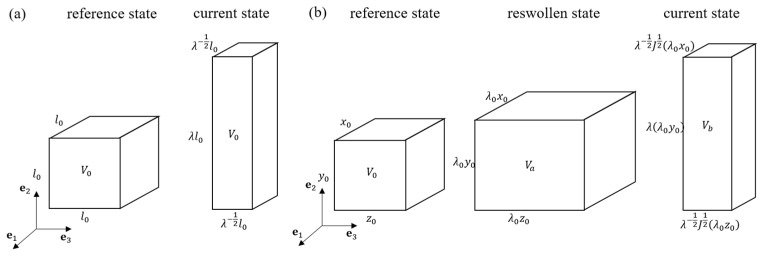
(**a**) Description of a uniaxial deformation process of a PAAm-Ca-alginate hydrogel along $e_{2}$ direction. The undeformed hydrogel is taken as the reference state, while the deformed state is denoted as the current state. (**b**) Description of the reswelling and stretching process of a pre-stretched PNaAMPS-PAAm hydrogel. The reference state represents the fully relaxed state of the pre-stretched hydrogel, with length $x_{0}$, $y_{0}$, and $z_{0}$ in each direction; after free reswelling, the hydrogel reaches the reswollen state, with length $\lambda_{0}x_{0}$, $\lambda_{0}y_{0}$, and $\lambda_{0}z_{0}$ in each direction. Then, the hydrogel is subjected to a stretch λ in $e_{2}$ direction, which is denoted as the current state.

DN hydrogels exhibit complex behaviors during deformation, encompassing water diffusion, viscoelasticity, and a Mullins-type response. As mentioned before, viscosity and water diffusion are characterized with different time scales, i.e., a DN hydrogel rarely exhibits a viscous response and water diffusion simultaneously [41]. Therefore, it is rational to investigate the impacts of these processes on the fatigue accumulation of DN hydrogels separately. In this work, we select two case studies with different loading conditions. For the first case, a PAAm-Ca-alginate hydrogel [17] is taken as the example to study the ionic-bond-breakage-induced fatigue damage with a negligible water diffusion effect. The other one employs a highly deformed PNaAMPS-PAAm hydrogel [21] to analyze the effect of water diffusion on the fatigue damage, in which a pre-stretch is applied to exclude the microdamage effect in the subsequent loading process. The loading conditions corresponding to the two cases are depicted in Figure 3, and a detailed description can be found in the following sections.

### 3.1. Microdamage and Viscoelastic Effects

**Figure 4:**
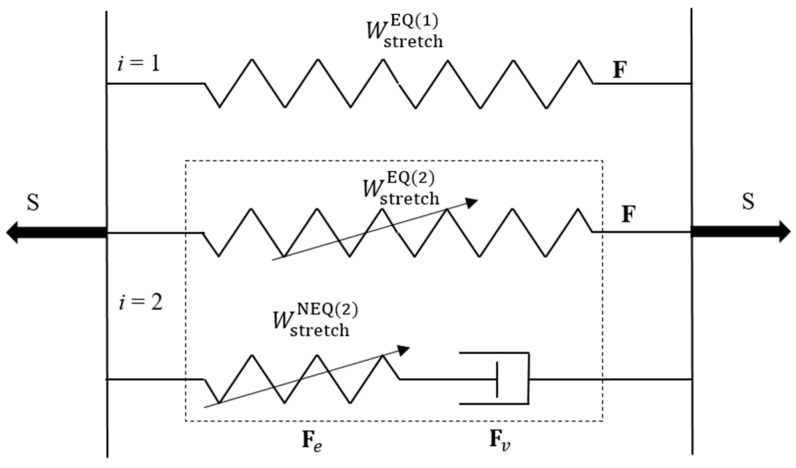
The parallel rheology model of a PAAm-Ca-alginate hydrogel [17], consisting of an intact spring, a damageable spring (a spring with an arrow), and a Maxwell element with a damageable spring and a dashpot.

Owing to the negligible water diffusion effect during the deformation, we take the swelling part of the deformation gradient as $F_{s}=I$, thereby $F=F_{m}$. According to Mao et al. [17], the double-network PAAm-Ca-alginate hydrogel is idealized to be completely incompressible, i.e., det(**F**) = 1. The rheology model of this DN hydrogel is illustrated in Figure 4, in which *i* = 1 represents the covalently crosslinked primary network (PAAm network) with long chains, while *i* = 2 represents the ionically crosslinked sacrificial network (Ca-alginate network) with short chains. As shown in this figure, three micromechanisms are adopted to model the mechanical response of this DN hydrogel [17]. The first micromechanism is taken to model the elastic response of the primary network, the second one represents the distributed microdamage of the sacrificial network accounting for the elasticity with a Mullins-type effect, and the third one represents the viscoelasticity of the sacrificial network due to intermolecular energetic bond-stretching.

The first and second micromechanisms are associated with an equilibrium response, while the third micromechanism is associated with a non-equilibrium response. Without losing generality, the same format of energy density functions could be taken for different micromechanisms following the work of Mao et al. [17]. For the first and second micromechanisms, the strain energy density functions $W_{stretch}^{EQ\left(1\right)}\left(F\right)$ and $W_{stretch}^{EQ\left(2\right)}\left(F,\xi^{EQ}\right)$ take the Gent model [23], while that of the third micromechanism $W_{stretch}^{NEQ\left(2\right)}\left(F_{e},\xi^{NEQ}\right)$ is described by the Hencky’s strain energy function [64,65], i.e.,

(39a) $$W_{\mathrm{stretch}}^{\mathrm{EQ}\left(1\right)}\left(F\right)=-\frac{G^{\mathrm{EQ}\left(1\right)}J_{\lim}^{\left(1\right)}}{2}\ln\left(1-\frac{I_{1}-3}{J_{\lim}^{\left(1\right)}}\right)$$

(39b) $$W_{\mathrm{stretch}}^{\mathrm{EQ}\left(2\right)}\left(F,\xi^{\mathrm{EQ}}\right)=-\frac{G^{\mathrm{EQ}\left(2\right)}J_{\lim}^{\left(2\right)}}{2}\ln\left(1-\frac{I_{1}-3}{J_{\lim}^{\left(2\right)}}\right)$$

(39c) $$W_{\mathrm{stretch}}^{\mathrm{NEQ}\left(2\right)}\left(F_{e},\xi^{\mathrm{NEQ}}\right)=G^{\mathrm{NEQ}\left(2\right)}\sum_{j=1}^{3}{\left(\ln\lambda_{j}^{e}\right)}^{2}+\frac{1}{2}\left(K-\frac{2}{3}G^{\mathrm{NEQ}\left(2\right)}\right){\left(\sum_{j=1}^{3}\ln\lambda_{j}^{e}\right)}^{2}\;$$

$G^{EQ\left(i\right)}$ and $G^{NEQ\left(i\right)}$ represent the equilibrium and the non-equilibrium shear moduli of the *i*th polymer network, $J_{\lim}^{\left(i\right)}$ denotes the chain stretchability limit of the *i*th network, and $\xi^{\mathrm{EQ}}$ and $\xi^{\mathrm{NEQ}}$ represent the internal variables characterizing the microdamage of the sacrificial network (*i* = 2) for the equilibrium and the non-equilibrium response. For the second micromechanism, the Mullins effect is realized by unzipping the ionic crosslinks in the sacrificial network. Based on the network alternation theory [25,26], the maximum effective distortional stretch in history was adopted by Mao et al. [17] as the equilibrium internal variable $\xi^{\mathrm{EQ}}$, i.e.,

(40) $$\xi^{\mathrm{EQ}}={\bar{\lambda }}_{\max}\left(t\right)=\underset{\eta \in \left[0,t\right]}{\max}\left[\bar{\lambda }\left(\eta \right)\right]\;\;\mathrm{with}\;\bar{\lambda }=\frac{1}{\sqrt{3}}\sqrt{\mathrm{tr}\left(C\right)}$$

Considering the microdamage effect on the material properties, the variation of the shear modulus $G^{EQ\left(2\right)}$ and the chain stretchability limit $J_{\lim}^{\left(2\right)}$ is described as

(41) $$G^{\mathrm{EQ}\left(2\right)}=G_{0}^{\mathrm{EQ}\left(2\right)}f\left({\bar{\lambda }}_{\max}\right),J_{\lim}^{\left(2\right)}=J_{\lim0}^{\left(2\right)}g\left({\bar{\lambda }}_{\max}\right)$$

where $G_{0}^{\mathrm{EQ}\left(2\right)}$ and $J_{\lim0}^{\left(2\right)}$ are the initial shear modulus and the chain stretchability limit, characterizing the chain density and the chain length of the undeformed sacrificial network. $f\left({\bar{\lambda }}_{\max}\right)$ and $g\left({\bar{\lambda }}_{\max}\right)$ should fulfill the following requirements:

(42a) $$f\left(1\right)=1,g\left(1\right)=1$$

(42b) $$f\left({\bar{\lambda }}_{\max}\right)g\left({\bar{\lambda }}_{\max}\right)=1$$

(42c) $$\frac{\partial f\left({\bar{\lambda }}_{\max}\right)}{\partial {\bar{\lambda }}_{\max}}<0,\frac{\partial g\left({\bar{\lambda }}_{\max}\right)}{\partial {\bar{\lambda }}_{\max}}>0$$

Equation (42a) describes the initial (undamaged) condition of the sacrificial network. Equation (42b) comes from the mass conservation of the sacrificial network, demonstrating that with the unzipping of the ionic crosslinks, the newly formed long chains are all active to contribute to the elasticity of the sacrificial network. The inequalities in (42c) satisfies the dissipation inequality (17a), implying that the chain stretchability limit $J_{\lim}^{\left(2\right)}$ increases and the shear modulus $G^{\mathrm{EQ}\left(2\right)}$ decreases with the microdamage of the sacrificial network. To fulfill the above requirements, $f\left({\bar{\lambda }}_{\max}\right)$ and $g\left({\bar{\lambda }}_{\max}\right)$ were prescribed in different formats in the literature [24]. Particularly, $f\left({\bar{\lambda }}_{\max}\right)$ and $g\left({\bar{\lambda }}_{\max}\right)$ were set by Mao et al. [17] as

(43) $$f\left({\bar{\lambda }}_{\max}\right)={\left(a+\left(1-a\right){\bar{\lambda }}_{\max}\right)}^{-b},g\left({\bar{\lambda }}_{\max}\right)={\left(a+\left(1-a\right){\bar{\lambda }}_{\max}\right)}^{b}$$

with 1 > *a* > 0 and *b* > 0 obtained through data fitting with experimental results.

Considering the coupled Mullins effect and viscoelastic response, an interaction between the intermolecular energetic bond-stretching (the third micromechanism) and the microdamage of the sacrificial network (the second micromechanism) is considered [17]. To account for such an interaction, the non-equilibrium internal variable $\xi^{\mathrm{NEQ}}$ describing the third micromechanism should be determined on the basis of $\xi^{\mathrm{EQ}}={\bar{\lambda }}_{\max}$, i.e.,

(44) $$\xi^{\mathrm{NEQ}}=\varphi {\bar{\lambda }}_{\max}$$

where $\varphi \in \left[0,1\right]$ evolves to characterize a fraction of the extent of the microdamage in the sacrificial network. By definition, φ is the percentage of the microdamage that affects the intermolecular energetic bond-stretching out of the total microdamage, assessing the interaction degree between the intermolecular energetic bond-stretching and the microdamage of the sacrificial network. According to the work of Mao et al. [17], the evolution of φ depends on the level of the effective distortional stretch $\bar{\lambda }$ and its rate $\dot{\bar{\lambda }}$, as

(45) $$\dot{\varphi }=\left\{\begin{array}{ll} A\left(1-\varphi \right)\dot{\bar{\lambda }} & \;\mathrm{if}\;\bar{\lambda }={\bar{\lambda }}_{\max}\;\mathrm{and}\;\dot{\bar{\lambda }}>0\;\left(1\mathrm{st}\;\mathrm{loading}\right)\\ A\varphi \dot{\bar{\lambda }} & \;\mathrm{if}\;\bar{\lambda }\leq {\bar{\lambda }}_{\max}\;\mathrm{and}\;\dot{\bar{\lambda }}<0\;\left(\mathrm{unloading}\right)\\ A\left[{\left(\frac{\bar{\lambda }-1}{{\bar{\lambda }}_{\max}-1}\right)}^{2}-\varphi \right]\dot{\bar{\lambda }} & \;\mathrm{if}\;\bar{\lambda }<{\bar{\lambda }}_{\max}\;\mathrm{and}\;\dot{\bar{\lambda }}>0\;\left(\mathrm{reloading}\right) \end{array}\right.$$

with A > 0 as a material constant, which can be determined from data fitting of the mechanical response of the polymer. A detailed explanation on the evolution of φ can be found in work by Mao et al. [17]. The viscoelasticity in the third micromechanism is affected by the internald variable $\xi^{\mathrm{NEQ}}=\varphi {\bar{\lambda }}_{\max}$, i.e., the shear modulus $G^{NEQ\left(2\right)}$ and the viscous flow resistance *s* vary in a form of

(46a) $$G^{\mathrm{NEQ}\left(2\right)}=G_{0}^{\mathrm{NEQ}\left(2\right)}+H_{g}{\left(\varphi {\bar{\lambda }}_{\max}\right)}^{r}$$

(46b) $$s=s_{0}+H_{s}{\left(\varphi {\bar{\lambda }}_{\max}\right)}^{q}$$

where $\left\{\begin{array}{cccccc} G_{0}^{\mathrm{NEQ}\left(2\right)} & H_{g} & r & s_{0} & H_{s} & q \end{array}\right\}$ in Equation (46) are all positive-valued parameters, as listed in Table 1.

When the hydrogel is subjected to uniaxial stretching along the **e**_2_ direction, as shown in Figure 3a, the deformation gradient follows $F=\mathrm{diag}\left(\lambda^{-\frac{1}{2}},\lambda ,\lambda^{-\frac{1}{2}}\right)$. For the sacrificial network, the elastic and the viscous deformation gradients are thus expressed as $F_{e}=\mathrm{diag}\left[\lambda_{e}^{-\frac{1}{2}},\lambda_{e},\lambda_{e}^{-\frac{1}{2}}\right]$ and $F_{v}=\mathrm{diag}\left[\lambda_{v}^{-\frac{1}{2}},\lambda_{v},\lambda_{v}^{-\frac{1}{2}}\right]$, respectively. Considering zero stress in the two lateral directions, i.e., *S*_11_ = *S*_33_ = 0, the expressions of the nominal stress *S*_22_ in the **e**_2_ direction are given as

(47) $$S_{22}=S_{22}^{\mathrm{EQ}\left(1\right)}+S_{22}^{\mathrm{EQ}\left(2\right)}+S_{22}^{\mathrm{NEQ}\left(2\right)}$$

with

(48a) $$S_{22}^{\mathrm{EQ}\left(1\right)}=G^{\mathrm{EQ}\left(1\right)}{\left(1-\frac{\lambda^{2}+2\lambda^{-1}-3}{J_{\lim}^{\left(1\right)}}\right)}^{-1}\left(\lambda -\lambda^{-2}\right)$$

(48b) $$S_{22}^{\mathrm{EQ}\left(2\right)}=G^{\mathrm{EQ}\left(2\right)}{\left(1-\frac{\lambda^{2}+2\lambda^{-1}-3}{J_{\lim}^{\left(2\right)}}\right)}^{-1}\left(\lambda -\lambda^{-2}\right)$$

(48c) $$S_{22}^{\mathrm{NEQ}\left(2\right)}=3G^{\mathrm{NEQ}\left(2\right)}\lambda^{-1}\ln\lambda^{e}$$

Due to the negligible water diffusion effect during the deformation, the mixing-induced configurational stress $\Sigma_{n}^{\mathrm{mix}}$ keeps constant with $d\Sigma_{n}^{\mathrm{mix}}=0$. According to the damage criteria in Equation (37), the mixing makes no contribution to the fatigue damage in this case. Therefore, the total fatigue damage is merely induced by the stretching of the polymer networks, i.e., $\Sigma_{n}={\left(\Sigma_{n}^{\mathrm{str}}\right)}^{ND}$, which can be computed through Equation (35). Substituting the strain energy density functions listed in Table 1 and the derived nominal stresses into Equation (35), the network configurational stresses ${\left(\Sigma_{n}\right)}_{i}$ are determined as

(49a) $$\begin{array}{l} {\left(\Sigma_{n}\right)}_{1}={\left(\Sigma_{n}\right)}_{3}=-\frac{G^{\mathrm{EQ}\left(1\right)}J_{\lim}^{\left(1\right)}}{2}\ln\left(1-\frac{\lambda^{2}+2\lambda^{-1}-3}{J_{\lim}^{\left(2\right)}}\right)-\frac{G^{\mathrm{EQ}\left(2\right)}J_{\lim}^{\left(2\right)}}{2}\ln\left(1-\frac{\lambda^{2}+2\lambda^{-1}-3}{J_{\lim}^{\left(2\right)}}\right)\\ +\frac{3}{2}G^{\mathrm{NEQ}\left(2\right)}{\left(\ln\lambda_{e}\right)}^{2} \end{array}$$

(49b) $$\begin{array}{ll} {\left(\Sigma_{n}\right)}_{2} & =G^{\mathrm{EQ}\left(1\right)}\left[-\frac{J_{\lim}^{\left(1\right)}}{2}\ln\left(1-\frac{\lambda^{2}+2\lambda^{-1}-3}{J_{\lim}^{\left(1\right)}}\right)-{\left(1-\frac{\lambda^{2}+2\lambda^{-1}-3}{J_{\lim}^{\left(1\right)}}\right)}^{-1}\left(\lambda^{2}-\lambda^{-1}\right)\right]\\ & +G^{\mathrm{EQ}\left(2\right)}\left[-\frac{J_{\lim}^{\left(2\right)}}{2}\ln\left(1-\frac{\lambda^{2}+2\lambda^{-1}-3}{J_{\lim}^{\left(2\right)}}\right)-{\left(1-\frac{\lambda^{2}+2\lambda^{-1}-3}{J_{\lim}^{\left(2\right)}}\right)}^{-1}\left(\lambda^{2}-\lambda^{-1}\right)\right]\\ & +3G^{\mathrm{NEQ}\left(2\right)}\left[\frac{1}{2}{\left(\ln\lambda -\ln\lambda_{v}\right)}^{2}-\left(\ln\lambda -\ln\lambda_{v}\right)\right] \end{array}$$

It can be seen that the values of ${\left(\Sigma_{n}\right)}_{1}$ and ${\left(\Sigma_{n}\right)}_{3}$ are always positive under the uniaxial stretching condition, as expressed in Equation (49a). According to the damage criteria Equation (37), the fatigue accumulation in this case is merely linked to the configurational stress ${\left(\Sigma_{n}\right)}_{2}$ in the **e**_2_ direction. The incremental format of ${\left(\Sigma_{n}\right)}_{2}$ is expressed as

(50) $$d{\left(\Sigma_{n}\right)}_{2}=d{\left(\Sigma_{n}\right)}_{2}^{\mathrm{EQ}}+d{\left(\Sigma_{n}\right)}_{2}^{\mathrm{NEQ}}$$

with

(51a) $$d{\left(\Sigma_{n}\right)}_{2}^{\mathrm{EQ}}=\sum_{i=1}^{2}G^{\mathrm{EQ}\left(i\right)}\left[\eta^{\left(i\right)}\left(-\lambda -2\lambda^{-2}\right)-\frac{2}{J_{\lim}^{\left(i\right)}}{\left(\eta^{\left(i\right)}\right)}^{2}\lambda {\left(\lambda -\lambda^{-2}\right)}^{2}\right]d\lambda$$

(51b) $$\begin{array}{ll} d{\left(\Sigma_{n}\right)}_{2}^{\mathrm{NEQ}} & =3G^{\mathrm{NEQ}\left(2\right)}\lambda^{-1}\left(\ln\lambda -\ln\lambda_{v}-1\right)d\lambda +\left[-3G^{\mathrm{NEQ}\left(2\right)}\lambda_{v}^{-1}\left(\ln\lambda -\ln\lambda_{v}-1\right)\right]d\lambda_{v}\\ & +3rH_{g}{\left({\bar{\lambda }}_{\max}\right)}^{r}{\left(\varphi \right)}^{r-1}\left[\frac{1}{2}{\left(\ln\lambda -\ln\lambda_{v}\right)}^{2}-\left(\ln\lambda -\ln\lambda_{v}\right)\right]d\varphi \end{array}$$

where $\eta^{\left(i\right)}={\left(1-\frac{\lambda^{2}+2\lambda^{-1}-3}{J_{\lim}^{\left(i\right)}}\right)}^{-1}$.

**Figure 5:**
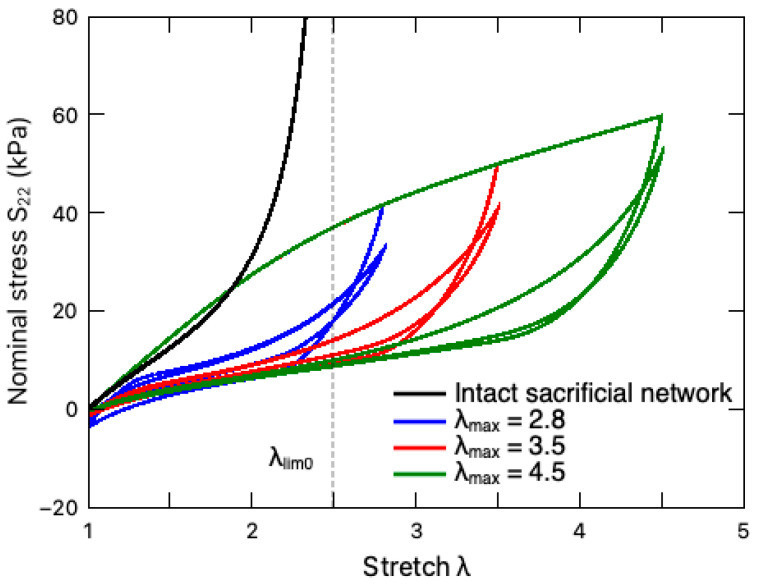
Multicycle responses of a PAAm-alginate hydrogel under different stretching amplitudes (maximum stretch ratio) at the rate of 8.8 min^−1^, which are denoted by colored solid lines. The black line represents the response of the DN hydrogel with an intact sacrificial network, i.e., $G^{EQ\left(2\right)}=G_{0}^{EQ\left(2\right)},{\;J}_{lim}^{\left(2\right)}=J_{lim0}^{\left(2\right)}$. The dashed vertical line indicates the stretching limit $\lambda_{lim0}$, where $\underset{\lambda \to \lambda_{\lim0}}{\lim}\left(I_{1}-3\right)=J_{lim0}^{\left(2\right)}$.

The multicycle responses of the PAAm-Ca-alginate hydrogel under different stretching amplitudes (maximum stretch ratio) are illustrated in Figure 5. For comparison purposes, the response of this DN hydrogel with an intact sacrificial network, i.e., $G^{EQ\left(2\right)}=G_{0}^{EQ\left(2\right)}$ and $J_{lim}^{\left(2\right)}=J_{lim0}^{\left(2\right)}$, is also plotted in this figure. It can be observed that the unzipping of the ionic crosslinks enables the DN hydrogel to exceed the original stretching limit $\lambda_{lim0}$, circumventing the stress stiffening around $\lambda_{lim0}$ [24]. In addition, such a crosslink breakage gives rise to the Mullins effect, where the stress in the first loading cycle is much larger than those in the subsequent cycles with respect to the same deformation. With the increasing number of loading cycles, the DN hydrogel eventually reaches a steady-state deformation cycle exhibiting hysteresis due to the viscosity of the material.

**Figure 6:**
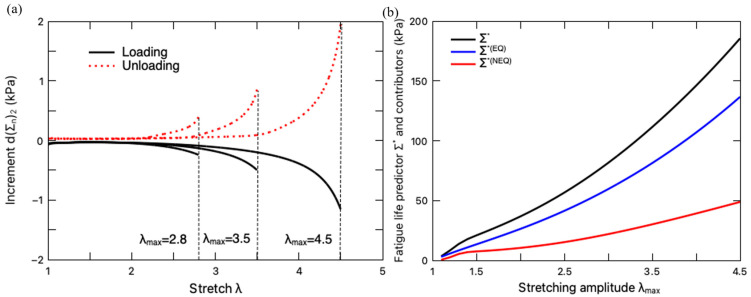
(**a**) Variation of the increment in configurational stress $d{\left(\Sigma_{n}\right)}_{2}$ with stretching. (**b**) Variation of fatigue life predictor $\Sigma^{*}$ and its contributors with stretching amplitude (loading rate: 8.8 min^−1^).

To assess the fatigue damage, the increment in the configurational stresses and the resultant predictor should be calculated in a steady-state loading cycle. Based on the steady-state cycle responses (Figure 5), the fatigue damage of the hydrogel under different stretching amplitudes is evaluated according to the damage criteria in Equation (37). The increment in the configurational stress $d{\left(\Sigma_{n}\right)}_{2}$ in the loading and unloading processes is plotted in Figure 6a. It can be seen that owing to the negative value of ${d\left(\Sigma_{n}\right)}_{2}$, the fatigue damage is merely accumulated in the loading process. Correspondingly, the fatigue life predictor $\Sigma^{*}$ of the hydrogel can be calculated on the loading path of a steady-state deformation cycle, which is plotted in Figure 6b with the variation of the stretching amplitude $\lambda_{max}$. It is clearly demonstrated that with the increase in the stretching amplitude in a loading cycle, more fatigue damage will be induced. To help elucidate this result, the fatigue damage due to the equilibrium deformation $\Sigma^{*\left(EQ\right)}$ and the non-equilibrium deformation $\Sigma^{*\left(NEQ\right)}$ are also plotted in this figure. The equilibrium part consists of the fatigue damage from the elastic response of the primary network and the sacrificial network, while the non-equilibrium part characterizes the fatigue damage accumulated through the viscoelastic response of the hydrogel due to the rate-dependent intermolecular energetic bond stretching. As expected, the increase in the stretching amplitude magnifies the total fatigue damage and its two contributors.

**Figure 7:**
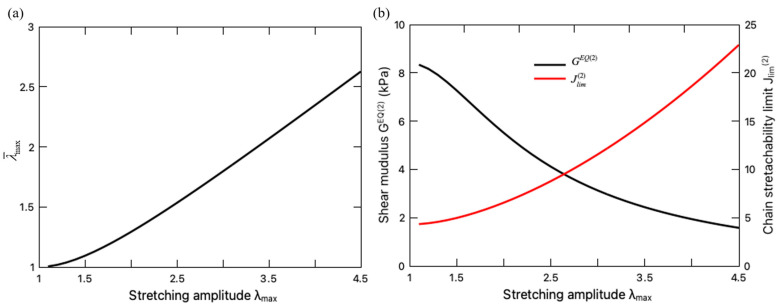
(**a**) Evolution of the microdamage level ${\bar{\lambda }}_{max}$ of the sacrificial network with stretching amplitude; (**b**) Variation of shear modulus $G^{EQ\left(2\right)}$ (left axis) and chain stretchability limit $J_{lim}^{\left(2\right)}$ (right axis) with stretching amplitude.

**Figure 8:**
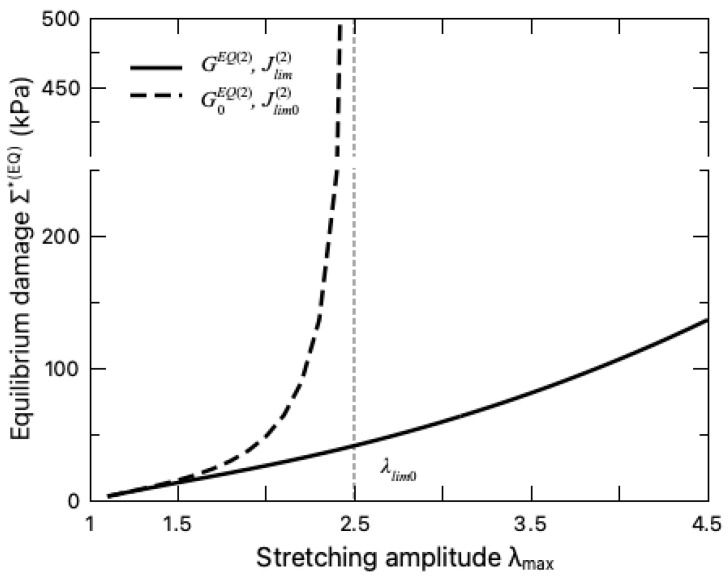
Variation of equilibrium damage $\Sigma^{*(EQ)}$ with stretching amplitude. The solid line represents the damaged sacrificial network with varying parameters $G^{EQ(2)}$ and $J_{lim}^{\left(2\right)}$, while the dashed line represents the case where the sacrificial network is intact, i.e., $G^{EQ\left(2\right)}(t)=G_{0}^{EQ(2)}$, $J_{lim}^{\left(2\right)}(t)=J_{lim0}^{\left(2\right)}$. The dashed vertical line indicates the stretching limit $\lambda_{lim0}$.

When assessing the equilibrium part $\Sigma^{*\left(EQ\right)}$ of the fatigue damage, the unzipping of the crosslinks in the sacrificial network needs to be taken into account, which is characterized by the variation of the internal variable ${\bar{\lambda }}_{max}$ (Figure 7a) and the material parameters, i.e., $G^{EQ\left(2\right)}$ and $J_{lim}^{\left(2\right)}$ (Figure 7b). Figure 7a indicates that with the increase in the stretching amplitude $\lambda_{max}$, the sacrificial network sustains more microdamage ${\bar{\lambda }}_{max}$. With more ionic crosslinks unzipped, the sacrificial network becomes softer with a decreasing shear modulus $G^{EQ\left(2\right)}$ and a larger stretchability limit $J_{lim}^{\left(2\right)}$. To further demonstrate the effect of the ionic-crosslink breakage on the fatigue damage, the values of $\Sigma^{*\left(EQ\right)}$ for the DN hydrogel with damaged and intact sacrificial network are compared in Figure 8, where the intact sacrificial network is realized by keeping the material parameters as constant during the entire deformation cycle, i.e., $G^{EQ\left(2\right)}\left(t\right)=G_{0}^{EQ\left(2\right)},J_{lim}^{\left(2\right)}\left(t\right)=J_{lim0}^{\left(2\right)}$. It can be seen from this figure that the microdamage effect on the fatigue damage is negligible when the hydrogel is undergoing small stretching as expected. When the stretching amplitude becomes larger, the breakage of the ionic crosslinks takes effect and releases the constraint of stretching limit $\lambda_{lim0}$ on the DN hydrogel, enhancing its resistance to the fatigue characterized by a lower $\Sigma^{*\left(EQ\right)}$. This finding is consistent with the widely recognized toughening mechanisms of DN hydrogels in the literature, i.e., the unzipping of crosslinks dissipates mechanical energy such that less energy drives crack nucleation and propagation, thereby preventing the advance of crack and toughening the DN hydrogel [5,37].

**Figure 9:**
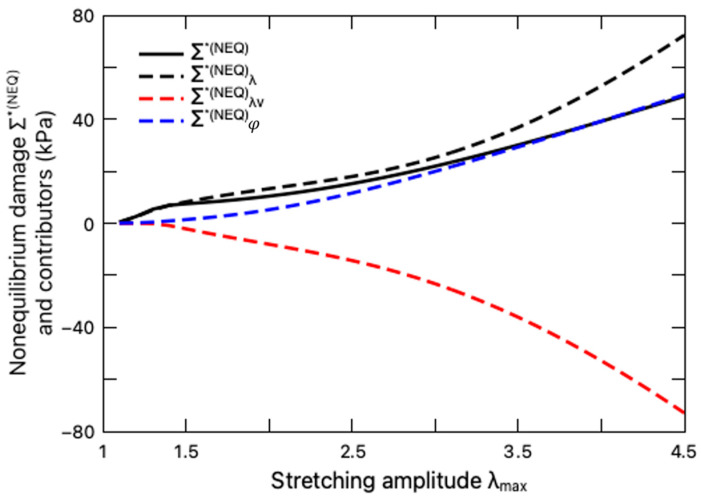
Variation of non-equilibrium damage $\Sigma^{*\left(NEQ\right)}$ (solid black line) and its contributors with stretching amplitude. The contributors from stretch λ, viscous stretch $\lambda_{v}$, and interaction φ are denoted by dashed black, red, and blue lines, respectively.

We further explore the impact of the intermolecular energetic bond-stretching on the fatigue damage of the hydrogel through the non-equilibrium damage $\Sigma^{*\left(NEQ\right)}$, as depicted in Figure 9 with the variation of the stretching amplitude. Calculated from Equation (49b) and determined through the damage criterion Equation (37), the non-equilibrium damage $\Sigma^{*\left(NEQ\right)}$ is a sum of three constituents, namely, the elastic part ${\left(\Sigma^{*\left(NEQ\right)}\right)}_{\lambda }$, the viscous part ${\left(\Sigma^{*\left(NEQ\right)}\right)}_{\lambda_{v}}$, as well as the interaction part ${\left(\Sigma^{*\left(NEQ\right)}\right)}_{\varphi }$, which are also plotted in Figure 9 for comparison. It is demonstrated that both the deformation λ and the interaction degree φ contribute to the non-equilibrium damage $\Sigma^{*\left(NEQ\right)}$ with positive values of ${\left(\Sigma^{*\left(NEQ\right)}\right)}_{\lambda }$ and ${\left(\Sigma^{*\left(NEQ\right)}\right)}_{\varphi }$, while the viscous deformation $\lambda_{v}$ mitigates the damage, as evidenced by the negative value of ${\left(\Sigma^{*\left(NEQ\right)}\right)}_{\lambda_{v}}$, which can be interpreted as energy dissipation due to viscosity and thus hinders the crack nucleation. This explanation is also coincident with the viscous effect on fatigue damage of elastomers [42].

**Figure 10:**
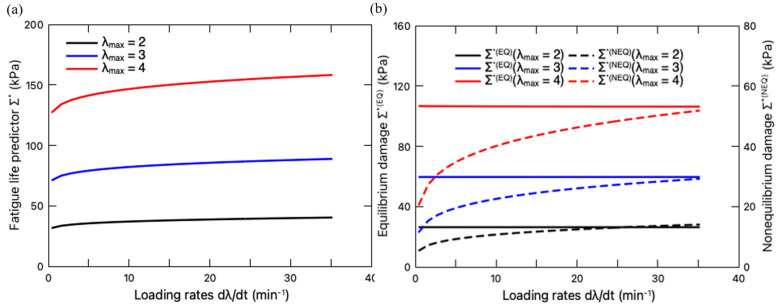
(**a**) Variation of fatigue life predictor $\Sigma^{*}$ with loading rate when the stretching amplitude is fixed; (**b**) Variation of equilibrium damage $\Sigma^{*(EQ)}$ (left axis) and non-equilibrium damage $\Sigma^{*(NEQ)}$ (right axis) with loading rate. The equilibrium portion is denoted by solid line, while the non-equilibrium portion is denoted by dashed line.

Then, we study the loading rate effect on the fatigue damage. When the stretching amplitude $\lambda_{max}$ is fixed, the total fatigue damage $\Sigma^{*}$ of the hydrogels under different loading rates is depicted in Figure 10a, with its equilibrium and non-equilibrium contributions plotted in Figure 10b. With fixed stretching amplitude $\lambda_{max}$, the fatigue damage $\Sigma^{*}$ increases with the increase in the loading rate, while reaching a plateau when the loading rate is sufficiently high. Due to the fixed maximum deformation, the microdamage ${\bar{\lambda }}_{max}$ keeps identical regardless of the loading rates, leading to an unchanged $\Sigma^{*\left(EQ\right)}$, as shown in Figure 10b. Therefore, the variation of $\Sigma^{*}$ only results from its non-equilibrium part $\Sigma^{*\left(NEQ\right)}$, which can be attributed to the impact of loading rate on the evolution of both the viscous deformation $F_{v}\;$(Equation (19)) and the interaction φ (Equation (45)). Overall, the increase in the values of $\Sigma^{*\left(NEQ\right)}$ can be explained as that less energy is dissipated through the viscoelastic response at a higher loading rate. On the other side, more energy will contribute to the crack nucleation. This observation is consistent with the results from the tearing test of PAAm-Ca-alginate hydrogels [36]. It showed that the energy release rate, i.e., the driving force of crack extension, increased with the loading rate. Also, Liu et al. [41] provided a prediction on the fatigue crack growth of viscous DN hydrogels, pointing out that the driving force of crack propagation increased with the increase in loading rates.

**Figure 11:**
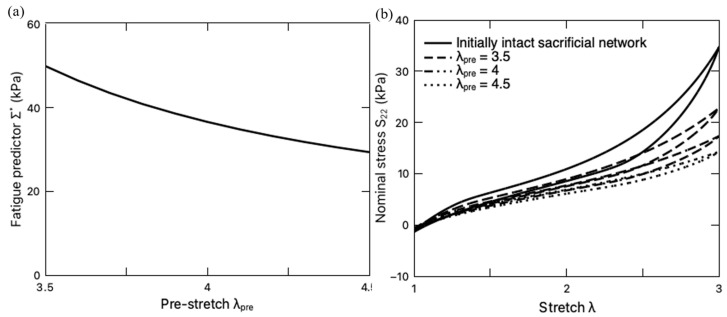
(**a**) Variation of fatigue life predictor $\Sigma^{*}$ with pre-stretch ratio $\lambda_{pre}$. (**b**) Steady-state stress–stretch curves of the PAAm-Ca-alginate hydrogel subjected to different pre-stretch ratios.

It should be mentioned that the previous discussions have focused on the fatigue damage of the PAAm-Ca-alginate hydrogels with an initially intact sacrificial network. However, it was found in the literature [66] that the PAAm-Ca-alginate hydrogel with a pre-stretch $\lambda_{pre}$ is capable of maintaining high resilience when the subsequent stretching is within the range of the resilient domain, i.e., $\lambda <\lambda_{pre}$. When $\lambda >\lambda_{pre}$, the sacrificial network continuously breaks and toughens the material, increasing the fracture toughness of the DN hydrogel. Inspired by this work, we further investigate the pre-stretching effect on fatigue damage. The pre-stretch ratio $\lambda_{pre}$ ranges from 3.5 to 4.5, while the subsequent cyclic deformation is set as $\lambda_{max}=3,$ which falls in the so-called resilient domain. With a fixed loading rate of 8.8 min^−1^, the variation of $\Sigma^{*}\;$ with the pre-stretch ratio $\lambda_{pre}$ is plotted in Figure 11a. It is observed that pre-stretching mitigates the total fatigue damage $\Sigma^{*}$. The steady-state stress–stretch response curves under such loading conditions are also plotted in Figure 11b in comparison with the case that the hydrogel is deformed with an initially intact sacrificial network. It is clearly demonstrated that pre-stretching makes the hydrogel softer, i.e., lower stress in comparison to that without pre-stretching. The reduction in the fatigue damage can be attributed to the softer sacrificial network with a lower shear modulus $G^{EQ\left(2\right)}$ and higher chain stretchability limit $J_{lim}^{\left(2\right)}$ (Figure 7b). In summary, the pre-stretching treatment endows DN hydrogels with higher fatigue resistance (lower $\Sigma^{*}$) in their resilient domain ($\lambda_{max}<\lambda_{pre}$).

### 3.2. Ultimate Swelling Effect

In the previous section, we limited our attention to the conditions with negligible a water diffusion effect. However, it has been observed in the literature that pre-stretching of DN hydrogels can induce a reswelling phenomenon [19]. Moreover, water diffusion also takes effect during the deformation under some loading conditions [21]. In this section, we will focus on the fatigue damage of a highly pre-stretched PNaAMPS-PAAm hydrogel undergoing uniaxial tension under equilibrium swelling. As shown in Figure 3b, we take the fully relaxed state of a pre-stretched hydrogel as the reference state, the fully reswollen state as the intermediate state, and the uniaxially stretched state as the current state. In the stretching process, the hydrogel is always equilibrating with water, i.e., μ=0. The deformation gradient **F** from the reference state to the current state follows

(52) $$F=\mathrm{diag}\left[\Lambda_{1},\Lambda_{2},\Lambda_{3}\right]$$

with

(53) $$\begin{array}{c} \Lambda_{2}=\lambda_{0}\lambda \\ \Lambda_{1}=\Lambda_{3}=\lambda_{0}\lambda^{-\frac{1}{2}}J^{\frac{1}{2}} \end{array}$$

Here, $\lambda_{0}={\left(V_{a}/V_{0}\right)}^{\frac{1}{3}}$ is the reswelling stretch, λ is the imposed mechanical stretch in **e**_2_ direction on the reswollen hydrogel, and $J=V_{b}/V_{a}$ represents the volume change from the reswollen state to the stretched state. Through the pre-stretching treatment, the short chains in the sacrificial network have been damaged; it is thus ensured that no chain breakage occurs during the subsequent stretching. Therefore, the response of the two polymer networks can be idealized as micromechanism 1, i.e., pure elastic response. To simulate the response of a PNaAMPS-PAAm hydrogel under uniaxial stretching with the consideration of equilibrium swelling, we adopt the constitutive model proposed by Imaoka et al. [21] in which the total free energy density *W* is composed of three parts, i.e., the stretching energy density *W*_stretch_, the mixing energy density *W*_mix_ between the two polymer networks and water, and the mixing energy *W*_ion_ between the mobile ions and water [52]. The stretching energy adopts the extended Gent model [21], i.e.,

(54) $$W_{\mathrm{stretch}}=-c_{10}J_{\lim}\ln\left(1-\frac{I_{1}-3}{J_{\lim}}\right)+c_{20}{\left(I_{1}-3\right)}^{2}$$

where $c_{10}$ and $c_{20}$ are material constants, and $I_{1}=\Lambda_{2}^{2}+2\Lambda_{1}^{2}$ is the first invariant of the deformation tensor. The mixing energy *W*_mix_ between the two polymer networks and water is described by using the Flory–Huggins lattice model [52,67] with the expression of

(55) $$W_{mix}=nkT\left[\phi_{f}\ln\phi_{f}+\chi_{12}\phi_{1}\phi_{2}+\chi_{1f}\phi_{1}\phi_{f}+\chi_{2f}\phi_{2}\phi_{f}\right]$$

Here, *n* denotes the total number of lattices of the hydrogel system, $v_{f}$ is the volume of each lattice, and thus the total volume of the hydrogel is $V_{b}/V_{0}=nv_{f}$ [67]. $\phi_{i}$ represents the volume fraction of each species *i* in the system (*i* = 1, 2 for polymer networks and *i* = *f* is for the water) with $\sum \phi_{i}=1$, and $\chi_{jk}$ denotes the Flory–Huggins interaction parameter between the species *j* and *k* (*j* and *k* = 1, 2, *f*). The volume fraction $\phi_{i}$ (*i* = 1, 2) is calculated from $\phi_{i}=\phi_{i0}J^{-1}$, with $\phi_{i0}$ being the volume fraction measured in the reswollen state [21]. The general expression of the mixing energy *W*_ion_ between the ions and water is given as [68,69]

(56) $$W_{\mathrm{ion}}=kT\left[C_{+}\left(\ln\frac{C_{+}}{c_{+}^{ref}V_{b}/V_{0}}-1\right)+C_{-}\left(\ln\frac{C_{-}}{c_{-}^{ref}V_{b}/V_{0}}-1\right)\right]$$

where $c_{+}^{ref}$ and $c_{-}^{ref}$ represent the initial concentration of the positive and negative mobile ions in the reference state, and $C_{+}$ and $C_{-}$ are the nominal concentration of the positive and the negative mobile ions, respectively. For this PNaMPA-PAAm hydrogel, Na^+^ is introduced as the fixed charge in the PAMPS network from the preparation process. Since the subsequent reswelling and stretching occur in the environment of pure water, the number of Na^+^ in the PAMPS network keeps constant, and there is no mobile Na^+^ in the water, i.e., $C_{+}=0$. Considering that only the mobile counterions contribute to the ionic energy, $C_{-}=c_{-}^{ref}\frac{V_{b}}{V_{0}}$. Let *α* be the ratio of the mobile counterions to the total counterions in the PNaAMPS network, and the nominal concentration of the mobile counterions is further written as ${\;C}_{-}=\frac{\alpha \phi_{1}}{v_{1}}\frac{V_{b}}{V_{0}}$. Hence, the expression of *W*_ion_ from Equation (56) reduces to

(57) $$W_{\mathrm{ion}}=-kT\frac{\alpha \phi_{1}}{v_{1}}\frac{V_{b}}{V_{0}}$$

In terms of the Flory–Rehner theory [51,52], the total swelling pressure of the hydrogel is the sum of the elastic pressure $\Pi_{\mathrm{stretch}}$, the mixing pressure $\Pi_{\mathrm{mix}}$, and the ionic pressure $\Pi_{\mathrm{ion}}$, i.e.,

(58) $$\Pi_{\mathrm{gel}}=\Pi_{\mathrm{stretch}}+\Pi_{\mathrm{mix}}+\Pi_{\mathrm{ion}}$$

with these pressures determined by $\Pi_{k}=-\frac{\partial W_{k}}{\partial \left(V_{b}/V_{0}\right)}$ [67]. Correspondingly, the expressions of these pressures are determined as

(59a) $$\Pi_{\mathrm{stretch}}=-2\Lambda_{2}^{-1}\left[\frac{c_{10}J_{\lim}}{J_{\lim}-I_{1}+3}+2c_{20}\left(I_{1}-3\right)\right]$$

(59b) $$\Pi_{\mathrm{mix}}=-\frac{kT}{v_{f}}\left[\ln\left(1-\phi_{1}-\phi_{2}\right)+\phi_{1}+\phi_{2}-\chi_{12}\phi_{1}\phi_{2}+\chi_{1f}\phi_{1}\left(\phi_{1}+\phi_{2}\right)+\chi_{2f}\phi_{2}\left(\phi_{1}+\phi_{2}\right)\right]$$

(59c) $$\Pi_{\mathrm{ion}}=kT\frac{\alpha \phi_{1}}{v_{1}}$$

When a hydrogel is at its swelling equilibrium state, the total swelling pressure $\Pi_{\mathrm{gel}}$ is taken to be zero, leading to

(60) $$\begin{array}{c} -2\Lambda_{2}^{-1}\left[\frac{c_{10}J_{\lim}}{J_{\lim}-I_{1}+3}+2c_{20}\left(I_{1}-3\right)\right]+kT\frac{\alpha \phi_{1}}{v_{1}}\\ -\frac{kT}{v_{f}}\left[\ln\left(1-\phi_{1}-\phi_{2}\right)+\phi_{1}+\phi_{2}-\chi_{12}\phi_{1}\phi_{2}+\chi_{1f}\phi_{1}\left(\phi_{1}+\phi_{2}\right)+\chi_{2f}\phi_{2}\left(\phi_{1}+\phi_{2}\right)\right]=0 \end{array}$$

The nominal stress in the **e**_2_ direction is derived based on the definition of Equation (25), i.e.,

(61) $$S_{22}=2\Lambda_{2}\left[\frac{c_{10}J_{\lim}}{J_{\lim}-I_{1}+3}+2c_{20}\left(I_{1}-3\right)\right]-\left(\Pi_{\mathrm{mix}}+\Pi_{\mathrm{ion}}\right)\Lambda_{1}^{2}$$

According to Equation (35), the network configurational stress $\Sigma_{n}$ consists of the non-dissipative part of the stretching-induced configurational stress ${\left(\Sigma_{n}^{\mathrm{str}}\right)}^{ND}$ and the mixing-induced configurational stress $\Sigma_{n}^{\mathrm{mix}}$, i.e., $\Sigma_{n}={\left(\Sigma_{n}^{\mathrm{str}}\right)}^{ND}+\Sigma_{n}^{\mathrm{mix}}$. For the PNaMPS-PAAm hydrogel undergoing a uniaxial tension under equilibrium swelling, ${\left(\Sigma_{n}^{\mathrm{str}}\right)}^{ND}=\Sigma_{n}^{\mathrm{str}}$, which is computed as

(62) $$\Sigma_{n}^{\mathrm{str}}=W_{\mathrm{stretch}}I-F^{T}S_{n}^{\mathrm{str}}$$

The mixing-induced configurational stress $\Sigma_{n}^{\mathrm{mix}}$ is derived as

(63) $$\Sigma_{n}^{mix}=\left(W_{\mathrm{mix}}+\Pi_{\mathrm{mix}}\Lambda_{2}\Lambda_{1}^{2}\right)I+\left(W_{\mathrm{ion}}+\Pi_{\mathrm{ion}}\Lambda_{2}\Lambda_{1}^{2}\right)I$$

From Equations (57) and (59c), it is determined that $W_{\mathrm{ion}}+\Pi_{\mathrm{ion}}\Lambda_{2}\Lambda_{1}^{2}=0$. Correspondingly, the mixing contribution to the configurational stress is reduced to

(64) $$\Sigma_{n}^{\mathrm{mix}}=\left(W_{\mathrm{mix}}+\Pi_{\mathrm{mix}}\Lambda_{2}\Lambda_{1}^{2}\right)I$$

Particularly, the stretching-induced configurational stress ${\left(\Sigma_{n}^{\mathrm{str}}\right)}_{2}$ in the **e**_2_ direction is expressed as

(65) $$\begin{array}{cc} {\left(\Sigma_{n}^{\mathrm{str}}\right)}_{2} & =c_{10}J_{\lim}\left[-\ln\left(\frac{J_{\lim}-\lambda_{0}^{2}\left(\lambda^{2}+2J\lambda^{-1}\right)+3}{J_{\lim}}\right)-\frac{2\lambda_{0}^{2}\lambda^{2}}{J_{\lim}-\lambda_{0}^{2}\left(\lambda^{2}+2J\lambda^{-1}\right)+3}\right]\\ & +c_{20}\left[\lambda_{0}^{2}\left(-3\lambda^{2}+2J\lambda^{-1}\right)-3\right]\left[\lambda_{0}^{2}\left(\lambda^{2}+2J\lambda^{-1}\right)-3\right] \end{array}$$

with its incremental format given as

(66) $$\begin{array}{cc} d{\left(\Sigma_{n}^{\mathrm{str}}\right)}_{2}= & \left\{\begin{array}{c} 2\lambda_{0}^{2}c_{10}J_{\lim}\left[\frac{-\lambda -J\lambda^{-2}}{J_{\lim}-\lambda_{0}^{2}\left(\lambda^{2}+2J\lambda^{-1}\right)+3}-\frac{2\lambda_{0}^{2}\left(\lambda^{3}-J\right)}{{\left(J_{\lim}-\lambda_{0}^{2}\left(\lambda^{2}+2J\lambda^{-1}\right)+3\right)}^{2}}\right]\\ +2c_{20}\lambda_{0}^{2}\left[-2\lambda_{0}^{2}\left(3\lambda^{3}+J+2J^{2}\lambda^{-3}\right)+6\left(\lambda +J\lambda^{-2}\right)\right] \end{array}\right\}d\lambda \\ + & \left\{\begin{array}{c} 2\lambda_{0}^{2}c_{10}J_{\lim}\left[\frac{\lambda^{-1}}{J_{\lim}-\lambda_{0}^{2}\left(\lambda^{2}+2J\lambda^{-1}\right)+3}-\frac{2\lambda_{0}^{2}\lambda }{{\left(J_{\lim}-\lambda_{0}^{2}\left(\lambda^{2}+2J\lambda^{-1}\right)+3\right)}^{2}}\right]\\ +4c_{20}\lambda_{0}^{2}\lambda^{-1}\left[\lambda_{0}^{2}\left(-\lambda^{2}+2J\lambda^{-1}\right)-3\right] \end{array}\right\}dJ \end{array}$$

Meanwhile, the mixing-induced configurational stress ${\left(\Sigma_{n}^{\mathrm{mix}}\right)}_{i}\;(i=1,2,3)$ is specified as

(67) $${\left(\Sigma_{n}^{\mathrm{mix}}\right)}_{i}=\frac{kT}{v_{f}}\lambda_{0}^{3}\left[\begin{array}{l} -\left(\phi_{10}+\phi_{20}\right)\ln\left(1-\phi_{10}J^{-1}-\phi_{20}J^{-1}\right)-\left(\phi_{10}+\phi_{20}\right)+2\chi_{12}\phi_{10}\phi_{20}J^{-1}\\ +\chi_{1f}\phi_{10}-2\chi_{1f}\phi_{10}\left(\phi_{10}+\phi_{20}\right)J^{-1}+\chi_{2f}\phi_{20}-2\chi_{2f}\phi_{20}\left(\phi_{10}+\phi_{20}\right)J^{-1} \end{array}\right]$$

with its incremental format

(68) $$d{\left(\Sigma_{n}^{\mathrm{mix}}\right)}_{i}=\left\{\frac{kT}{v_{f}}\lambda_{0}^{3}J^{-2}\left[\begin{array}{l} -\frac{{\left(\phi_{10}+\phi_{20}\right)}^{2}}{1-\phi_{10}J^{-1}-\phi_{20}J^{-1}}-2\chi_{12}\phi_{10}\phi_{20}+2\chi_{1f}\phi_{10}\left(\phi_{10}+\phi_{20}\right)\\ +2\chi_{2f}\phi_{20}\left(\phi_{10}+\phi_{20}\right) \end{array}\right]\right\}dJ$$

It should be mentioned that the configurational stresses in **e**_1_ and **e**_3_ directions are not presented here as they will not contribute to the fatigue damage. The material parameters of the hydrogel are listed in Table 2, which will be used for the following simulations.

**Figure 12:**
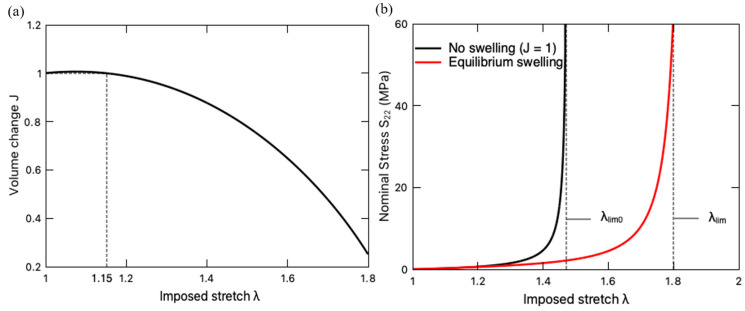
(**a**) Volume change *J* of PNaMPS-PAAm hydrogel subjected to uniaxial stretching under equilibrium swelling condition. (**b**) Stress–stretch curve of PNaMPS-PAAm hydrogel under both equilibrium swelling and fast loading without swelling effect.

Figure 12a shows the variation in volume change of the hydrogel with the imposed stretch *λ* when the PNaMPS-PAAm hydrogel is under a uniaxial stretching, while Figure 12b presents the corresponding stress–stretch curve. It is found in Figure 12a that the hydrogel slightly swells (*J* > 1) under a relatively small stretching at the beginning of loading, then undergoes a deswelling behavior (*J* < 1) at a larger stretch. Such an extension-induced deswelling behavior is attributed to the limiting stretch effect, i.e., the constraint of $\lambda_{lim}$ [70,71]. Meanwhile, this DN hydrogel exhibits a drying-induced softening, as exhibited by the stress–stretch curve in Figure 12b. When the imposed stretch exceeds a certain value of λ=1.15 (Figure 12a), deswelling occurs, and such a drying process induces a softening behavior, i.e., the lower stress value on the curve of the equilibrium swelling case in comparison to the fast-loading case without swelling effect, i.e., *J* = 1. In addition, the deswelling extends the stretching limit from the original $\lambda_{lim0}$ to a new $\lambda_{lim}$, allowing the pre-stretched DN hydrogel to undergo a larger uniaxial stretch. The extension-induced deswelling and the drying-induced softening are the symptoms of the inverse mechanical-swelling coupling defined by Imaoka et al. [21].

**Figure 13:**
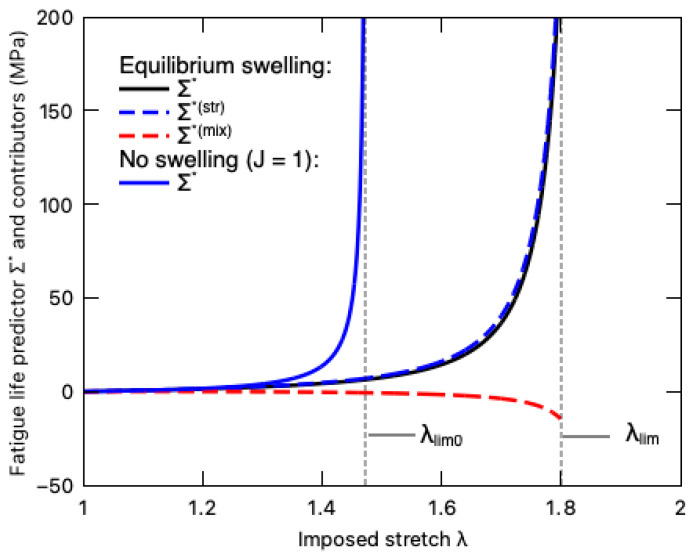
Variation of fatigue life predictor $\Sigma^{*}$ (solid black line) and its stretching-induced $\Sigma^{*(str)}$ (dashed blue line) and mixing-induced $\Sigma^{*(mix)}$ (dashed red line) contributors with imposed stretch λ when the PNaMPS-PAAm hydrogel undergoes equilibrium swelling. For comparison, the fatigue life predictor $\Sigma^{*}$ of this DN hydrogel undergoing fast loading without swelling effect (*J* = 1) is also plotted by the solid blue line.

The fatigue life predictor $\Sigma^{*}$ of the DN hydrogel under equilibrium swelling and its two contributors, i.e., the stretching-induced damage $\Sigma^{*(str)}$ and the mixing-induced damage $\Sigma^{*(mix)}$, is demonstrated in Figure 13. For comparison purposes, the fatigue damage predictor $\Sigma^{*}$ of the DN hydrogel undergoing fast loading without a swelling effect (J=1) is also plotted in this figure. Even though deswelling mitigates the total fatigue damage with the negative value of the mixing-induced contributor $\Sigma^{*(mix)}$ [43], the total fatigue damage $\Sigma^{*}$ is dominated by stretching, as observed by the nearly overlapping curves between $\Sigma^{*}$ and $\Sigma^{*(str)}$. Comparing the total fatigue predictor of the DN hydrogel during the equilibrium swelling and the fast-loading processes, we find that before the imposed stretch λ approaches the hydrogel’s original stretching limit $\lambda_{lim0}$, the hydrogel under the equilibrium swelling bears lower fatigue damage mainly due to the drying-induced softening. When the imposed stretch λ exceeds $\lambda_{lim0}$, the total fatigue damage accumulates very slowly until the stress stiffening occurs around the new stretching limit of the material. In other words, the inverse mechanical–swelling coupling behavior extends the stretching limit of DN hydrogels and enhances their fatigue resistance (lower $\Sigma^{*}$), whereas, since the inverse mechanical–swelling coupling was first experimentally reported in 2023 [21], there has been a lack of direct experimental validation on the fatigue damage, which could be a future research topic.

## 4. Conclusions

In this work, a fatigue life predictor based on the configurational mechanics framework is formulated to characterize the fatigue damage of DN hydrogels. With the proposed predictor, some fatigue mechanisms correlating to the microstructural change of the polymer networks have been revealed. The simulation results indicate that the breakage of the ionic crosslinks in the sacrificial network mitigates the fatigue damage of the DN hydrogels and improves their resistance to the fatigue crack nucleation. The results also reflect the loading rate effect on the fatigue damage, i.e., the DN hydrogels loaded under higher loading rates are more prone to fatigue crack nucleation due to less energy dissipation. In addition, the pre-stretching effects on the fatigue crack nucleation of DN hydrogels are also examined. Neglecting the water-diffusion effect, the pre-stretching treatment can improve the durability of the DN hydrogels within the resilient domain. While considering the equilibrium swelling condition of the pre-stretched DN hydrogel, it is shown that the total fatigue damage predominantly results from the stretching-induced part, which is lowered due to the drying-induced softening behavior. This work has attempted to unveil the fatigue mechanisms of DN hydrogels and interpret some experimental observations. It is expected to provide guidance on the design and optimization of loading profiles of hydrogel-based devices in practical applications.

## Figures and Tables

**Table 1:** Material parameters for the PAAm-Ca-alginate hydrogel [17].

*i*	Parameter	Value
1	$G^{\mathrm{EQ}\left(1\right)}$	2.03 kPa
	$J_{\lim}^{\left(1\right)}$	330
2	$G_{0}^{\mathrm{EQ}\left(2\right)}$	8.4 kPa
	$J_{\lim0}^{\left(2\right)}$	4.3
	*a*	0.196
	*b*	2
	$G_{0}^{\mathrm{NEQ}\left(2\right)}$	0.2 kPa
	H*_g_*	70.48 kPa
	*r*	1.3
	${\dot{\gamma }}_{0}$	1
	*m*	0.22
	*s*_0_	0.2 kPa
	H*_s_*	25.88 kPa
	*q*	1.34
	*A*	3

**Table 2:** Material parameters of the PNaMPS-PAAm hydrogel [21].

Parameter	Value
$\lambda_{0}$	2.2
$c_{10}$	45 kPa
$c_{20}$	−1.8 kPa
$J_{lim}$	14.1
$v_{f}$	0.03 nm^3^
$v_{1}$	0.2 nm^3^
$\chi_{12}$	0.015
$\chi_{1f}$	0.3
$\chi_{2f}$	0.45
$\phi_{10}$	0.0152
$\phi_{20}$	0.0723
*k*	1.370649 × 10^−23^ J/K
*T*	293 K
α	0.237

## Data Availability

Data are contained within the article.
